# Immune dysregulation caused by homozygous mutations in *CBLB*

**DOI:** 10.1172/JCI154487

**Published:** 2022-10-17

**Authors:** Erin Janssen, Zachary Peters, Mohammed F. Alosaimi, Emma Smith, Elena Milin, Kelsey Stafstrom, Jacqueline G. Wallace, Craig D. Platt, Janet Chou, Yasmeen S. El Ansari, Tariq Al Farsi, Najim Ameziane, Ruslan Al-Ali, Maria Calvo, Maria Eugenia Rocha, Peter Bauer, Nouriya Abbas Al-Sannaa, Nashat Faud Al Sukaiti, Abdullah A. Alangari, Aida M. Bertoli-Avella, Raif S. Geha

**Affiliations:** 1 Division of Immunology, Boston Children’s Hospital and Harvard Medical School, Boston, Massachusetts, USA.; 2 Department of Pediatrics, College of Medicine, King Saud University, Riyadh, Saudi Arabia.; 3 The Royal Hospital Muscat, Muscat, Oman.; 4 Centogene GmbH, Rostock, Germany.; 5 Johns Hopkins Aramco Health Care, Dhahran, Saudi Arabia.

**Keywords:** Immunology, Autoimmune diseases, Mast cells, T cells

## Abstract

CBL-B is an E3 ubiquitin ligase that ubiquitinates proteins downstream of immune receptors to downregulate positive signaling cascades. Distinct homozygous mutations in *CBLB* were identified in 3 unrelated children with early-onset autoimmunity, one of whom also had chronic urticaria. Patient T cells exhibited hyperproliferation in response to anti-CD3 cross-linking. One of the mutations, p.R496X, abolished CBL-B expression, and a second mutation, p.C464W, resulted in preserved CBL-B expression. The third mutation, p.H285L in the SH2 domain of CBL-B, was expressed at half the normal level in the patient’s cells. Mice homozygous for the CBL-B p.H257L mutation, which corresponds to the patient’s p.H285L mutation, had T and B cell hyperproliferation in response to antigen receptor cross-linking. *Cblb^H257L^* mice had increased percentages of T regulatory cells (Tregs) that had normal in vitro suppressive function. However, T effector cells from the patient with the p.H285L mutation and *Cblb^H257L^* mice were resistant to suppression by WT Tregs. Bone marrow–derived mast cells from *Cblb^H257L^* mice were hyperactivated after FcεRI cross-linking, and *Cblb^H257L^* mice demonstrated exaggerated IgE-mediated passive anaphylaxis. This study establishes CBL-B deficiency as a cause of immune dysregulation.

## Introduction

The E3 ubiquitin ligase CBL-B is an important regulatory protein highly expressed in immune cells. CBL-B facilitates ubiquitination of receptor-activated signaling proteins, expediting their degradation, and plays an important role in peripheral T cell tolerance and curbing autoimmunity (1, 2).

In T cells, CBL-B expression is tightly regulated by signals downstream of the T cell receptor (TCR) and CD28 (2). Lymphocytes from *Cblb^–/–^* mice have increased proliferation and IL-2 production in response to antigen receptor cross-linking uncoupled from CD28 costimulation (3). Mast cells (MCs) from *Cblb^–/–^* mice are hyperactivated after FcεRI receptor engagement, with increased secretion of proinflammatory cytokines (4). Single-nucleotide polymorphisms in *CBLB* have been identified in type I diabetes (5), Graves’ disease (6), and multiple sclerosis (7). No published reports to our knowledge have identified *CBLB* variants as drivers of immune dysregulation. We report homozygous germline disease-causing variants in *CBLB* in 3 unrelated patients with early-onset autoimmunity.

## Results and Discussion

### Patient characteristics.

**Figure 1:**
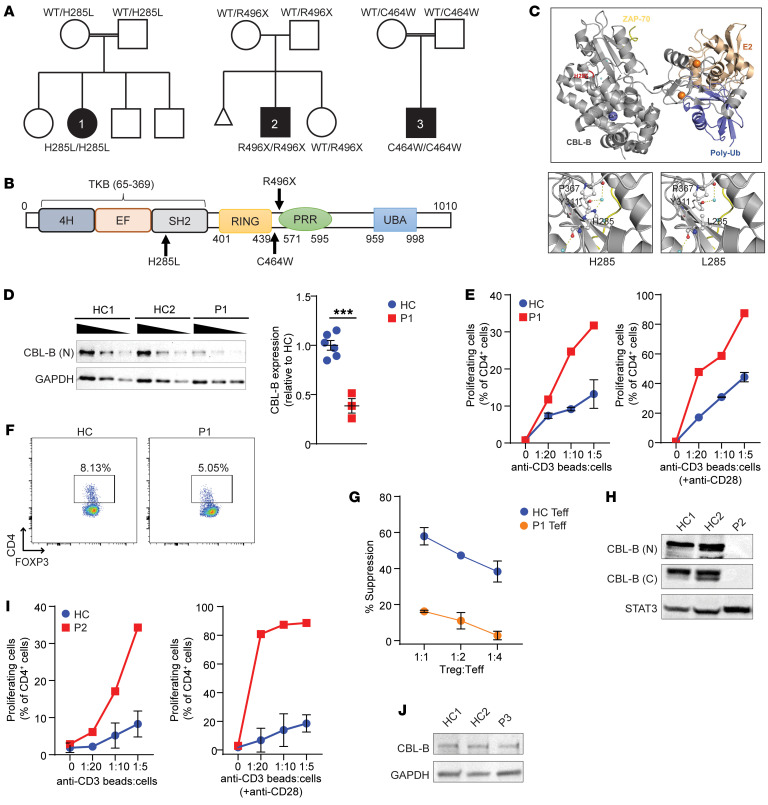
Homozygous *CBLB* mutations in the patients. (**A**) Pedigrees of P1, P2, and P3. (**B**) Linear map of CBL-B. The arrows indication locations of the patient mutations. (**C**) Ribbon diagram of the human CBL-B TKB and RING domains (aa 65–502, gray) in complex with an E2 ligase (brown), polyubiquitin (blue), and ZAP-70 peptide (yellow). Insets show potential local interactions between WT H285 (left) or mutant L285 (right). (**D**) Immunoblots of titrated lysates from EBV-transformed B cells from HC1, HC2, and P1 probed with an N-terminal antibody directed against CBL-B and anti-GAPDH (left). Densitometry of CBL-B bands normalized to GAPDH (right). One representative experiment out of 2 is shown. ***P < 0.001 by unpaired, 2-tailed Student’s *t* test. (**E**) Percentage of proliferating CD4^+^ T cells from 2 HCs and P1 following stimulation with anti-CD3 (left) and anti-CD3 plus anti-CD28 (right). (**F**) Representative plots of Tregs for HC and P1. (**G**) Suppression of proliferation of CD4^+^CD25^–^ Teffs from P1 and HCs by HC Tregs. Data shown are from 2 experiments. (**H**) Representative immunoblots of lysates from EBV-transformed B cells from HC1, HC2, and P2 probed with CBL-B and STAT3 antibodies. (**I**) Percentage of proliferating CD4^+^ T cells from 2 HCs and P2. (**J**) Representative immunoblots of lysates from EBV-transformed B cells from 2 HCs and P3 probed with CBL-B and GAPDH antibodies. Data in **D**, **E**, **G**, and **I** are presented as mean ± SEM.

Patient 1 (P1) is a 16-year-old girl born to Saudi first-cousin parents (Figure 1A). She developed hypothyroidism at 1 year old. At 6 years, she was diagnosed with type I diabetes and vitiligo and later developed recurrent urticaria. She gave no history of atopy. Serum IgE antibodies against environmental allergens were not detected. She had recurrent middle ear and lower respiratory tract infections. Intravenous immunoglobulin (IVIg) was started at 13 years old with improvement in her infections and chronic urticaria. A trial of abatacept did not improve her symptoms.

Peripheral blood T, B, and NK cell numbers were normal (Supplemental Table 1; supplemental material available online with this article; https://doi.org/10.1172/JCI154487DS1). Serum IgM, IgG, and IgA were low (Supplemental Table 1). Antibody titers after immunization with tetanus, diphtheria, and pneumococcal vaccines were nonprotective. Levels of IgG antibodies against 19 autoantigens, including the diabetes-associated autoantigens insulin and GAD65, were increased (Supplemental Figure 1). Her plasma, but not plasma from healthy controls (HCs), strongly activated normal basophils, suggesting that a serum activating factor or autoantibody underlies her chronic urticaria (Supplemental Table 1). Plasma levels of IL-6, TNF-α, IL-10, CXCL9, and CXCL10 were substantially elevated (Supplemental Table 1).

Patient 2 (P2) is an 11-year-old boy born to Saudi parents who reported no consanguinity (Figure 1A). He presented at 3 years old with fevers, lung infiltrates, hepatomegaly, and ascites responsive to corticosteroids, antibiotics, IVIg, and azathioprine. Targeted panel gene sequencing for hemophagocytic lymphohistiocytosis (HLH) mutations was negative. An infectious trigger for HLH could not be identified. Blood, stool, and pleural cultures, as well as serologies and antigen testing for CMV, EBV, HIV, hepatitis A and B, brucella, and aspergillus were negative. He was later diagnosed with hypothyroidism and type I diabetes. At 7 years old, he developed fevers with arthralgias and vomiting. Bone marrow aspiration revealed hemophagocytosis. He responded to treatment with steroids and azathioprine. He was recently admitted with fevers that were controlled by increasing his azathioprine dose. T, B, and NK cell numbers and immunoglobulin levels were unremarkable. Tetanus antibody titer was protective (Supplemental Table 1). IgG antibodies against 14 autoantigens, including insulin, were markedly increased (Supplemental Figure 1). Only modestly elevated plasma levels of the tested cytokines and chemokines were noted, possibly related to him being asymptomatic at the time of blood draw.

Patient 3 (P3) is a 4-year-old boy born to Omani first-cousin parents (Figure 1A). He developed autoimmune hemolytic anemia at 6 months old. He was diagnosed with idiopathic thrombocytopenia purpura (ITP) when 1 year old that was successfully treated with steroids and IVIg. He recently had a flare of ITP and was treated with a course of steroids and IVIg. Laboratory evaluation showed low hemoglobin and platelet count. T and B cell numbers were normal. IgM was borderline low. Antibody response to pneumococcal vaccination was nonprotective (Supplemental Table 1). Serum levels of IL-6, TNF-α, CXCL9, and CXCL10 were elevated (Supplemental Table 1). No plasma sample was available for analyzing antibodies to self-antigens.

### All 3 patients have homozygous disease-causing variants in CBLB.

Exome sequencing did not identify any relevant variants in known disease-causing genes in the patients. Three different homozygous variants in *CBLB* were identified. The variants were not present in gnomAD (v.2.1.1) nor our bio/databank of 60,234 individuals. Additional rare homozygous variants in the patients are in Supplemental Table 2.

P1 was homozygous for *CBLB* c.854A>T:p.H285L (NM_001321786.1). Both parents were heterozygous. The CBL-B H285 residue is within the SH2 binding subdomain (Figure 1B). This mutation is predicted to be deleterious (Supplemental Table 2). The SH2 subdomain is part of the tyrosine kinase binding (TKB) domain, which mediates the interaction of CBL-B with its substrates (8). Structural modeling indicated that H285L may distort a water binding pocket in CBL-B (Figure 1C). Immunoblotting of EBV-transformed B cell lysates from P1 and HCs with antibodies directed to the CBL-B N-terminus and C-terminus demonstrated that P1’s cells expressed the CBL-B H285L mutant at a level approximately 50% that of HCs (Figure 1D). CD4^+^ T cells from P1 had enhanced proliferation in response to anti-CD3 compared with controls (Figure 1E). Further increase in the proliferation was observed upon addition of anti-CD28 (Figure 1E). This proliferation profile is similar to T cells from *Cblb^–/–^* mice (3, 9). P1 also had a decreased percentage of peripheral T regulatory cells (Tregs) among CD4^+^ T cells (5.05% compared with 9.16% ± 1.46% in HCs) (Figure 1F). In addition, the proliferation of CD4^+^CD25^–^ Teffs from P1 was resistant to suppression by Tregs from HCs (Figure 1G).

P2 was homozygous for the nonsense variant *CBLB* c.1486C>T:p.R496X (Figure 1A). Both parents were heterozygous. Translation of the p.R496X CBL-B mutant was expected to be a 57 kDa truncated product. An antibody directed against the N-terminus of CBL-B failed to detect a truncated product in the EBV-B cells from P2 (Figure 1H). Similar to P1, CD4^+^ T cells from P2 hyperproliferated when stimulated with anti-CD3 and anti-CD3 plus anti-CD28 (Figure 1I).

P3 was homozygous for the missense variant c.1392C>G:p.C464W (Figure 1A); both parents were heterozygous (Figure 1A). Immunoblotting of the patient’s EBV-B cells demonstrated intact CBL-B protein expression (Figure 1J). Additional patient samples were not available for functional studies. The C464 residue is situated between the RING and PRR (Figure 1B). The region containing human CBL-B C464 is not available in the RCSB Protein Data Bank for modeling.

Early-onset autoimmunity and T cell hyperproliferation in response to CD3 ligation in 3 unrelated patients with distinct homozygous variants in CBL-B, one of which abolished protein expression, support a role for CBL-B deficiency in causing human immune dysregulation.

### Cblb^H257L^ mice display immune dysregulation.

**Figure 2:**
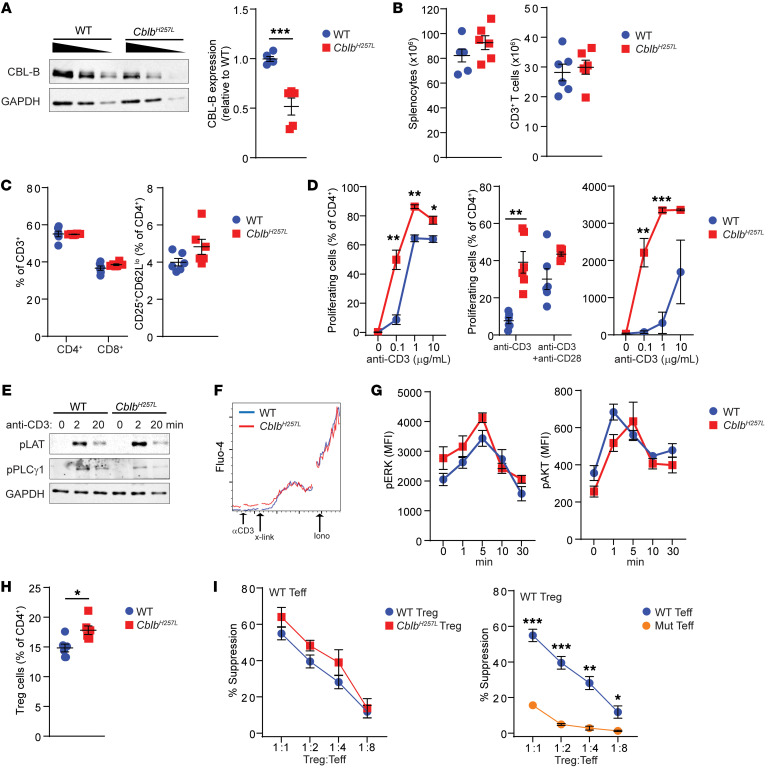
*Cblb^H257L^* T cells hyperproliferate in response to anti-CD3 and are resistant to Treg suppression. (**A**) Representative immunoblots of CBL-B and GAPDH expression in titrated T cell lysates (left). Densitometry of CBL-B bands normalized to GAPDH (right). (**B** and **C**) Numbers of splenocytes and CD3^+^ splenic T cells (**B**) and percentages of T cell subsets (**C**) in 8- to 10-week-old mice (*n =* 6 mice/group). (**D**) T cell proliferation (left) in response to anti-CD3, anti-CD3 plus anti-CD28 (middle), and IL-2 secretion (right) in response to anti-CD3. *n =* 3–6 mice/group. (**E**) T cells were activated with anti-CD3 for 2 and 20 minutes. Immunoblots for phospho-LAT, phospho-PLCγ1, and GAPDH. (**F**) T cells were stimulated with CD3 cross-linking followed by ionomycin. Calcium flux was measured by Fluo-4. One representative experiment out of 6 is shown. (**G**) T cells were stimulated with anti-CD3 for 0–30 minutes and phospho-ERK and phospho-AKT levels were determined by flow cytometry. (**H**) Percentages of Tregs in the spleen. (**I**) Suppression of proliferation of CD4^+^CD25^–^ WT Teffs by WT and *Cblb^H257L^* Tregs (left). Suppression of *Cblb^H257L^* and WT Teff proliferation by WT Tregs (right). One experiment out of 3 is shown for **A**, **D**, and **G** and out of 2 for **B**, **C**, **H**, and **I**. Data in **A**–**D** and **G**–**I** are presented as mean ± SEM. *P < 0.05; **P < 0.01; ***P < 0.001 by unpaired, 2-tailed Student’s *t* test.

The mouse CBL-B H257 residue is the correlate of human CBL-B H285. To ascertain whether P1’s *CBLB* p.H285L mutation predisposes to immune dysregulation, homozygous *Cblb* c.770A>T:p.H257L (*Cblb^H257L^*) mice were generated using CRISPR/Cas9 gene editing. Similar to EBV-B cells from P1, T cells from *Cblb^H257L^* mice expressed the mutant protein at levels approximately 50% that of WT (Figure 2A).

*Cblb^H257L^* mice were healthy with normal weight gain. At 1 year old, they did not display lymphadenopathy or organomegaly, and histologic studies did not reveal cellular infiltrates in their salivary glands, livers, or kidneys. Titers of autoantibodies reactive to HEp-2 cells were comparable between 1-year-old *Cblb^H257L^* and WT mice (data not shown). Autoantibodies and spontaneous autoimmune disease have been detected in one, but not the other of 2 independently generated lines of *Cblb^–/–^* mice (3, 10), suggesting that environmental and genetic factors play an important role in the development of autoimmune disease in CBL-B deficiency.

The numbers of splenocytes and splenic T cells, as well as the percentages of CD4^+^ cells, CD8^+^ cells, and activated CD4^+^ T cells (CD25^+^CD62L^lo^) in the spleen, were comparable between *Cblb^H257L^* mice and WT controls (Figure 2, B and C). CD4^+^CD25^–^ Teffs from *Cblb^H257L^* mice demonstrated hyperproliferation and increased IL-2 secretion after anti-CD3 stimulation (Figure 2D). Unlike WT T cells, they did not demonstrate increased proliferation upon addition of anti-CD28 (Figure 2D).

Despite hyperproliferation in response to anti-CD3 stimulation, *Cblb^H257L^* T cells demonstrated no increase in phosphorylation of LAT and PLC-γ1 (Figure 2E) and had normal intracellular calcium flux after CD3 cross-linking (Figure 2F), consistent with findings in T cells from *Cblb^–/–^* mice (3, 11). ERK1/2 and AKT phosphorylation after anti-CD3 stimulation were normal in *Cblb^H257L^* T cells (Figure 2G), as in *Cblb^–/–^* T cells (11).

*Cblb^H257L^* mice had an increased percentage of Tregs in the spleen compared with WT controls (Figure 2H). This contrasts with the lower percentage of Tregs in the blood of P1 (Figure 1F). This may be related to species differences, the use of medications, and/or disease-related complications in the patient. Tregs isolated from *Cblb^H257L^* mice and controls were equally effective at suppressing proliferation of WT Teffs stimulated with anti-CD3 and antigen-presenting cells (Figure 2I). *Cblb^H257L^* Teffs showed significant resistance to suppression by WT Tregs (Figure 2I). Increased IL-2 production by *Cblb^H257L^* Teffs may contribute to enhanced Teff proliferation/survival and resistance to Treg suppression, both also observed in *Cblb^–/–^* mice (12). Both Teff hyperproliferation and resistance to Treg suppression may contribute to the development of autoimmune disease in CBL-B–deficient patients.

**Figure 3:**
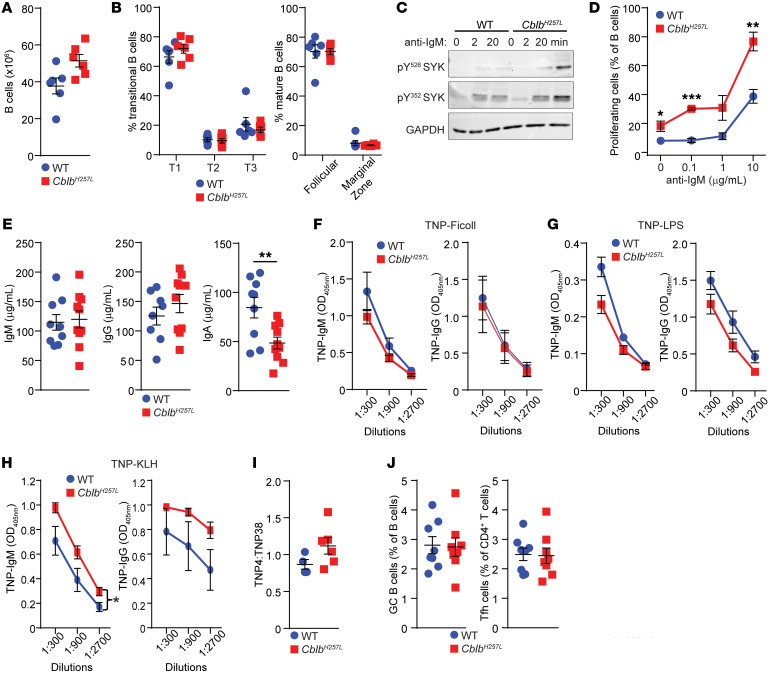
*Cblb^H257L^* B cells are hyperactivated with anti-IgM cross-linking. (**A** and **B**) Number of splenic B cells (**A**) and percentages of B cell subsets (**B**) in 8- to 10-week-old mice. *n =* 6 mice per group. (**C**) Immunoblots of phospho-SYK and GAPDH in B cells after stimulation with anti-IgM. (**D**) B cell proliferation in response to anti-IgM. *n =* 3 mice/group. (**E**) Serum IgM, IgG, and IgA from 8- to 10-week-old mice. *n =* 9–10 mice/group. (**F**–**I**) Serum IgM and IgG anti-TNP for mice immunized with TNP-Ficoll (**F**), TNP-LPS (**G**), and TNP-KLH (**H**), and affinity of TNP-IgG antibodies in TNP-KLH immunized mice (**I**). *n =* 4–6 mice/group. (**J**) Percentage of GC B cells and Tfh cells in the draining lymph nodes 10 days after immunization with TNP-KLH. One experiment out of 2 is shown for **A** and **B**, and out of 3 for **D** and **I**. Data in **A**, **B**, and **D**–**J** are presented as mean ± SEM. *P < 0.05; **P < 0.01; ***P < 0.001 by unpaired, 2-tailed Student’s *t* test (**D** and **E**) or 2-way ANOVA (**H**).

B cell numbers and subsets were comparable between *Cblb^H257L^* mice and controls (Figure 3, A and B). In *Cblb^H257L^* B cells, B cell receptor (BCR) ligation induced increased and prolonged phosphorylation of SYK at tyrosine residues 352 and 526, both important for SYK activation (13) (Figure 3C). They also demonstrated hyperproliferation after anti-IgM cross-linking compared with WT B cells (Figure 3D). Increased proliferation and SYK hyperphosphorylation after BCR cross-linking have been reported in *Cblb^–/–^* B cells (3).

Serum immunoglobulin concentrations have been reported to be normal in *Cblb^–/–^* mice (14). In one report, *Cblb^–/–^* mice were found to have increased antibody responses to immunization with T-independent and T-dependent antigens, the latter associated with increased germinal center (GC) B cells (15). However, in a recent study, GC B cells were found to be similar between *Cblb^–/–^* mice and WT controls after immunization with sheep red blood cells (16). Serum IgA, but not IgM or IgG, levels were decreased in *Cblb^H257L^* mice (Figure 3E). *Cblb^H257L^* mice mounted normal IgM and IgG responses to immunization with the T-independent antigens, trinitrophenyl-Ficoll (TNP-Ficoll) and TNP-LPS (Figure 3, F and G). Following immunization with the T-dependent antigen TNP–keyhole limpet hemocyanin (TNP-KLH), anti-TNP IgM levels were modestly increased in *Cblb^H257L^* mice, whereas anti-TNP IgG levels and affinity and the percentages of GC B cells and Tfh cells in the draining lymph nodes were not significantly different from controls (Figure 3, H–J). These results indicate that the augmented BCR signaling and B cell proliferation in *Cblb^H257L^* mice does not result in increased generation of GC B cells or antibody production by plasma cells, which no longer express the BCR.

### Cblb^H257L^ mice have MCs that are hyperresponsive to FcεRI cross-linking and demonstrate exaggerated IgE-mediated anaphylaxis.

**Figure 4:**
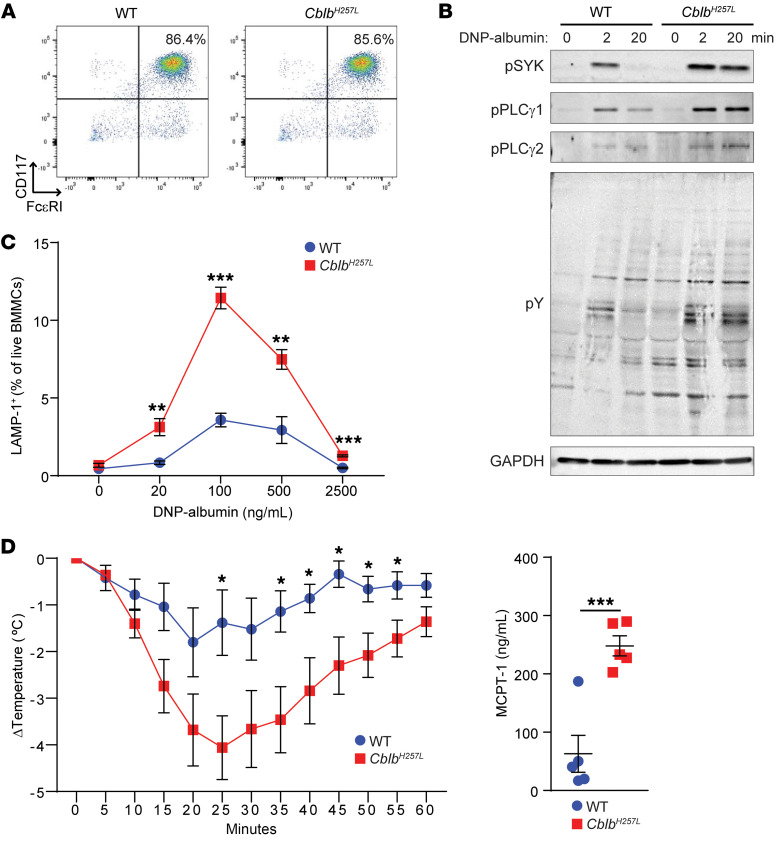
*Cblb^H257L^* BMMCs are hyperresponsive to FcεRI cross-linking, and *Cblb^H257L^* mice have exaggerated IgE-mediated anaphylaxis in response to oral antigen challenge. (**A**) CD117 and FcεRI expression on BMMCs. (**B**) Immunoblots of phospho-SYK, phospho-PLCγ1, phospho-PLCγ1, and total phospho-tyrosine (pY) of lysates from BMMCs stimulated by FcεRI cross-linking. (**C**) Surface LAMP-1 expression following FcεRI cross-linking. *n =* 5 mice/group. (**D**) Core temperature from 0–60 minutes (left) and serum MCPT-1 levels at 60 minutes (right) after challenge of mice passively sensitized with TNP-IgE and challenged by intragastric gavage with TNP-BSA. *n =* 5 mice/group. One representative experiment out of 2 shown in **B** and **D**, and out of 3 for **C**. Data in **C** and **D** are presented as mean ± SEM. *P < 0.05; **P < 0.01; ***P < 0.001 by unpaired, 2-tailed Student’s *t* test.

P1 developed chronic urticaria, suggesting MC hyperactivation. To examine the impact of the CBL-B H258L mutation on MC function, BMMCs were generated from *Cblb^H257L^* mice. *Cblb^H257L^* and control BMMCs demonstrated comparable CD117 and FcεRI expression (Figure 4A). To examine signaling events downstream of FcεRI, BMMCs were preincubated with dinitrophenyl-IgE (DNP-IgE) before crosslinking with DNP-albumin. *Cblb^H257L^* BMMCs demonstrated increased and sustained tyrosine phosphorylation of SYK, PLC-γ1, and PLC-γ2 (Figure 4B), similar to *Cblb^–/–^* BMMCs (4, 14). When BMMCs are activated, the lysosomal membrane protein LAMP-1 is mobilized to the cell surface. After FcεRI cross-linking, *Cblb^H257L^* BMMCs demonstrated an increase in LAMP-1 surface expression compared with WT BMMCs (Figure 4C).

Passive oral anaphylaxis was used to examine whether *Cblb^H257L^* MCs exhibit an exaggerated response to FcεRI cross-linking. *Cblb^H257L^* mice and controls were i.p. injected with TNP-IgE. Twenty-four hours later, TNP-BSA was administered by gavage. *Cblb^H257L^* mice had a substantial decrease in core body temperature compared with controls from 25 to 55 minutes after gavage with TNP-BSA (Figure 4D). One-hour after challenge, serum mast cell protease 1 (MCPT-1) levels were significantly higher in *Cblb^H257L^* mice compared with controls (Figure 4D). CBL-B ubiquitinates and degrades STAT6; consequently, CBL-B deficiency promotes the differentiation of IL-4–producing Th2 cells as well as IL-4 activation of MCs (17). Thus intrinsic as well as extrinsic effects may contribute to enhanced anaphylaxis in *Cblb^H257L^* mice.

P1, but not P2 or P3, has hypogammaglobulinemia and frequent infections. It is possible that P1 has an additional genetic variant(s) that underlies her hypogammaglobulinemia. Exome sequencing analysis did not reveal mutations in any known or candidate genes explaining the hypogammaglobulinemia in P1 (Supplemental Table 2).

Autoimmune diseases are usually thought to be triggered by environmental factors, especially infections in genetically susceptible individuals (18). *Cblb^H257L^* mice did not develop autoimmune disease under specific pathogen–free (SPF) conditions. There also has been variability in the development of autoimmunity in *Cblb^–/–^* mice (3, 10) that may be related to their housing conditions, microbiome, and/or the presence of modifier genes. Exposure to common murine pathogens by cohousing with pet-store mice has been a valuable tool for uncovering the immune dysregulation phenotype in a number of mouse models mimicking patient gene mutations (19). Additional experiments, including cohousing with pet-store mice, will be utilized to explore the development of autoimmunity in *Cblb^H257L^* mice.

In summary, we have identified 3 unrelated children with early-onset autoimmunity and homozygous mutations in *CBLB* predicted to affect protein function and/or expression. Like T cells from *Cblb^–/–^* mice (3, 10), CBL-B–deficient patients had hyperproliferation of their CD4^+^ T cells in response to anti-CD3. In addition, *Cblb^H257L^* mice, which mimic the mutation in P1, have hyperproliferation of CD4^+^ T cells and B cells in response to antigen receptor cross-linking and their Teffs were resistant to Treg suppression. The combination of T cell hyperproliferation, resistance to Treg suppression, and likely increased BCR signaling may contribute to the development of autoimmunity in CBL-B–deficient patients.

## Methods

See Supplemental Methods for a detailed description of all experimental procedures.

### Statistics.

Comparisons were analyzed for statistical significance using unpaired, 2-tailed Student’s *t* test and 2-way ANOVA to determine the *P* value using Prism software (GraphPad Software, Inc.). A *P* value of less than 0.05 was considered significant.

### Study approval.

All mouse studies were approved and performed in accordance with Boston Children’s Hospital Institutional Animal Research and Care Committee. Written informed consent for genetic testing, research studies, and storage of data and data processing was obtained from the parents.

## Author contributions

EJ, ZP, MFA, ES, EM, JGW, and YSEA performed the experiments and analyzed the data. KS, CDP, and JC generated the *Cblb^H275L^* mice. TAF, NFAS, NAAS, and AAA provided patient samples. NA, RAA, MC, MER, PB, and AMBA preformed the sequencing and exome analyses. EJ, AAA, AMBA, and RSG designed and supervised the research. EJ and RSG wrote the manuscript.

## Supplementary Material

Supplemental data

## Version 1 08/25/2022

In-Press Preview

## Version 2 10/17/2022

Electronic publication
